# Quality of life related to functional dependence, family functioning and social support in older adults^*^

**DOI:** 10.1590/1980-220X-REEUSP-2021-0482en

**Published:** 2022-05-27

**Authors:** Mayra Alejandra Mireles Alonso, Martha Elba Salazar Barajas, Jesús Alejandro Guerra Ordóñez, Hermelinda Ávila Alpirez, Jack Roberto Silva Fhon, Tirso Duran-Badillo

**Affiliations:** 1 Universidad Autónoma de Tamaulipas, Unidad Académica Multidisciplinaria Matamoros, Tamaulipas, México.; 2 Universidade de São Paulo, Escola de Enfermagem, Departamento de Enfermagem Médico Cirúrgica, São Paulo, SP, Brazil.

**Keywords:** Quality of Life, Activities of Daily Living, Family Relations, Social Support, Aged, Qualidade de Vida, Atividades Cotidianas, Relações Familiares, Apoio Social, Idoso, Calidad de Vida, Actividades Cotidianas, Relaciones Familiares, Apoyo Social, Anciano

## Abstract

**Objective:**

to determine the relationship between quality of life and functional dependence, family functioning and social support in older adults in northeastern Mexico.

**Method:**

this is a quantitative, descriptive and analytical study, with 205 older adults. The Barthel Index, Lawton and Brody Scale, APGAR test, Multidimensional Scale of Perceived Social Support and WHOQOL-BREF were applied.

**Results:**

the physical, psychological and social dimensions of quality of life are related to functional capacity, family functioning and social support (*p*< 0.05). Functional capacity and social support predict physical and psychological quality of life. Basic activities, family function and social support predict social quality of life. Instrumental activities and social support predict environmental quality of life.

**Conclusion:**

quality of life depends on functional independence, family function and positive perceived health. Independence in basic activities and adequate social support improve physical quality of life. Being independent in basic activities and social support improve psychological quality of life. When presenting a decrease in independence in instrumental activities, perceived quality of life decreases.

## RESUMEN

**Objetivo:**

determinar la relación entre la calidad de vida y la dependencia funcional, funcionamiento familiar y apoyo social en adultos mayores del noreste de México.

**Método:**

estudio cuantitativo, descriptivo y analítico, en 205 adultos mayores. Se aplicó índice de Barthel, Escala de Lawton y Brody, Test de APGAR, Escala Multidimensional de Apoyo Social Percibido y WHOQOL-BREF.

**Resultados:**

las dimensiones física, psicológica y social de la calidad de vida están relacionadas con capacidad funcional, funcionamiento familiar y apoyo social (*p*< 0,05). La capacidad funcional y apoyo social predicen la calidad de vida física y psicológica. Las actividades básicas, función familiar y apoyo social predicen la calidad de vida social; las actividades instrumentales y apoyo social predicen la calidad de vida ambiental.

**Conclusión:**

la calidad de vida depende de la independencia funcional, función familiar y percepción positiva de salud. La independencia en actividades básicas y adecuado apoyo social mejoran la calidad de vida física. Ser independiente en actividades básicas y el apoyo social mejoran la calidad de vida psicológica. Al presentar disminución de la independencia en actividades instrumentales, la percepción de la calidad de vida disminuye.

## RESUMO

**Objetivo:**

determinar a relação entre qualidade de vida e dependência funcional, funcionamento familiar e apoio social em idosos no nordeste do México.

**Método:**

estudo quantitativo, descritivo e analítico em 205 idosos. Foram aplicados o Índice de Barthel, a Escala de Lawton e Brody, o Teste APGAR, a Escala Multidimensional de Suporte Social Percebido e o WHOQOL-BREF.

**Resultados:**

as dimensões física, psicológica e social da qualidade de vida estão relacionadas à capacidade funcional, funcionamento familiar e suporte social (p < 0,05). A capacidade funcional e o suporte social predizem a qualidade de vida física e psicológica. Atividades básicas, função familiar e suporte social predizem a qualidade de vida social; atividades instrumentais e apoio social predizem a qualidade de vida ambiental.

**Conclusão:**

a qualidade de vida depende da independência funcional, da função familiar e da percepção positiva da saúde. A independência nas atividades básicas e o suporte social adequado melhoram a qualidade de vida física. Ser independente nas atividades básicas e no apoio social melhora a qualidade de vida psicológica. Ao apresentar diminuição da independência nas atividades instrumentais, a percepção de qualidade de vida diminui.

## INTRODUCTION

Aging is an inevitable phase of the life cycle, presenting with several age-related changes and is the phase of life in which functional dependence can occur more frequently^(1)^, as well as changes in family functioning and in perceived social support^(2)^, which can impact older adults’ quality of life.

It has been reported that functional dependence in older people in high-income countries is between 15 and 17%, and in low- and middle-income countries, between 3 and 16%. Meanwhile, in the Mexican population, it was indicated that 21.7% have limitations to perform Basic Activities of Daily Living and 5.1% are dependent^(3)^ and is an important predictor of mortality^(4)^. In this sense, the problem of functional dependence in older adults in Mexico is worrying, as projections for 2026 indicate that 18.9% of this population will suffer mild functional dependence, and 9.3%, severe functional dependence^(5)^.

Aging and dependence, closely associated, can cause changes in older adults’ quality of life^(6)^ and the need for family support to carry out their daily activities^(7)^. In Mexican culture, care takes place within families, which over time and physical, emotional and economic effort, in addition to changes they must make in their routine of life, and can culminate in family dysfunctions^(8)^.

Family functioning is a source of well-being and enjoyment of a satisfactory quality of life when it comes to families that value respect for older adults and manage the changes that occur with aging^(9)^. It has been reported that if older adults are in a functional family, their level of self-esteem, psychological health, personal and environmental relationships will be favored with an impact on their quality of life^(10)^.

Although it is true, in recent years it has contributed to the understanding of the phenomenon of quality of life in older adults and interventions have been proposed. The literature has shown that it is important to continue analyzing functionality, family and social support as variables that contribute to older adults’ quality of life^(11)^.

Nursing professionals need empirical knowledge for decision-making in primary care. However, previous studies have analyzed the relationship between quality of life and functional dependence^(6)^, quality of life with family functioning^(10)^ and quality of life with social support^(12)^, i.e., studies carried out separately. This shows a knowledge gap that explains the effect of functional dependence, family functioning and social support on quality of life. It is expected that the results can formulate nursing interventions aimed at guidance, education and prevention of events that affect older adults’ quality of life.

This study aimed to determine the relationship between quality of life and functional dependence, family functioning and social support in older adults in northeastern Mexico.

## METHOD

### Desing of Study

This is a cross-sectional, descriptive, analytical study with a quantitative approach.

### Local

The study was conducted at the “*Las Culturas*” Community Center belonging to Sanitary Jurisdiction No. 3, in the city of Heroica Matamoros, Tamaulipas, Mexico, which serves people of all age groups, and according to the last census carried out in 2018, approximately 1,200 older adults are served.

### Population and Sample

The sample size was determined using the WinEpi^©^ program for a population of 1,200 older adults, with a confidence level of 95%, an absolute error of 5% and an expected proportion of 20%, resulting in a total of 205 older adults. As the census is approximate and there is no sampling frame that allows randomization, subjects were identified by convenience sampling using the snowball technique.

Initially, they went to the Community Center where the first older adults were identified. Then, they indicated where more older adults cared for at the same Community Center could be found, who were located through home visits, and were asked to indicate where there were more people with the same characteristics following the same technique until completing the sample size.

### Inclusion and Exclusion Criteria

Older adults aged 60 years or older, residing in their family context, living with their family for at least six months and undergoing treatment at the Community Center were included. Older adults who did not have the ability to respond coherently to the questions, supported by the questioning of name, age and place of residence, were excluded.

### Data Collection

Information collection was between the months of January to July 2018 through interviews conducted in their family context and the following instruments were applied:

– Demographic profile: card with personal data such as age, sex, marital status, religion and membership in a social club and with chronic diseases.
– Barthel Index: validated for the Mexican population^(13)^, whose objective is to assess an individual’s capacity to carry out 10 Basic Activities of Daily Living (BADL) such as bath/shower, personal hygiene, dressing, use of the toilet, use of stairs, control of urine, control of feces, feeding, transfer to the chair and movement. The scale presents a score from zero to 100, with a higher score indicating less dependence.
– Lawton and Brody Scale: reliable and considered the gold standard at the international level in measuring Instrumental Activities of Daily Living (IADL)^(14)^ and assesses ability to use the phone, travel by motor vehicle to distant places, shop, control medication, manage finances, clean the house, do laundry, and prepare meals. It has a score from zero to eight points, with the higher the score indicating less dependence.
– Family APGAR test: allows identifying families with conflict problems or family dysfunctions, with five questions that explore adaptation, participation, gradient of personal resources, affection and resources. Each answer has a score that ranges from zero to four points, to then make a sum that ranges from zero to 10 points. The higher the score, the better the perceived family functioning is considered. The instrument’s internal consistency, in the Spanish version, has a Cronbach’s alpha of 0.84^(15)^.
– Multidimensional Scale of Perceived Social Support (MSPSS): identifies social support and consists of 12 statements grouped into three subscales referring to three sources of social support: family, friends and significant others. It consists of four response alternatives in Likert format, which include the alternatives and a rating: “almost never = 1”, “sometimes = 2”, “often = 3” and “almost always or always = 4”. For the interpretation, a sum is made, where the higher the score, the greater the perceived social support. This scale presents Cronbach’s alpha values greater than 0.80 for each of the three factors extracted^(16)^.
– WHOQOL-BREF: instrument created by the World Health Organization to measure quality of life, composed of 26 items. The first two questions are independent and investigate individuals’ general perceived quality of life and health, respectively, the remaining 24 assess four specific dimensions of quality of life: physical (items 3, 4, 10, 15, 16, 17 and 18), psychological (items 5, 6, 7, 11, 19 and 26), social (item 20, 21 and 22), and environmental (item 8, 9, 12, 13, 14, 23, 24 and 25).

Each question is rated on a scale of one to five. The higher the score, the higher the quality of life. The scores of the negative items (3, 4 and 26) are inverted, and the total scores obtained are converted into a scale from zero to 100 to establish comparisons between the domains, as they are composed of an unequal number of items, with Cronbach’s alpha of 0.89 for the full scale, and values greater than 0.70 for the dimensions^(17)^.

### Data Analysis and Treatment

Data were processed and analyzed in Statistical Package for the Social Sciences (SPSS) v. 20 for Windows. For sociodemographic aspects and main variables, descriptive statistics, frequencies and percentages were used for categorical variables, and for numerical variables, measures of dispersion were used, such as mean and standard deviation, as well as minimum and maximum values with 95% Confidence Intervals. Moreover, the Kolmogorov-Smirnov test with Lilliefors test was applied to identify normality in all study variables, being considered non- normal. Thus, Spearman’s correlation test was applied.

To respond to the general objective, a general linear multivariate contrast model was used, where age, sex, BADL and IADL dependence, family function and social support were placed as independent variables, and individuals’ general perceived quality of life and health, as well as the physical, psychological, social and environmental dimensions of quality of life were placed as dependent variables.

An inter-subject effects test was carried out to observe generally whether covariates as a whole have an effect on dependent variables and whether, in particular, any covariate has an effect on any dependent variable. Results were considered significant when p-value was less than 0.05 in each interaction. Likewise, through the Beta coefficient statistic, the mean effect of increasing covariates on dependent variables used was measured, whose actual value of the prediction or effect was considered to be within the limits of a 95% Confidence Interval.

### Ethical Aspects

The research project was assessed and authorized by the Research Ethics Committee of the Matamoros Multidisciplinary Academic Unit belonging to the *Universidad Autónoma de Tamulipas* (Opinion 090). Therefore, the study subject integrity was always taken care of, as established in the Declaration of Helsinki of the World Medical Association.

## RESULTS

According to the results on participants’ sociodemographic characteristics, it was possible to identify that 71.7% (ƒ = 147) corresponded to the female sex. Participants’ mean age was 70.30 years (*SD*= 7.44). As for marital status, 62.9% (*f*= 129) reported having a partner and the mean education was 4.23 years of study (*SD*= 3.07), where 18.0% (*f*= 37) reported not having education and 82.0% (*f*= 168) reported education between 1 and 15 years of study. Regarding other participants’ personal aspects, 82.0% (ƒ = 168) reported not belonging to a social club, 86.3% (ƒ = 177) had some religion and 76.6% (ƒ = 157) suffered from some chronic illness.

In relation to functional dependence, for BADL a mean of 94.12 (*SD*= 13.28) was obtained and for IADL a mean of 6.39 (*SD*= 2.29). In relation to family functioning, there was a mean of 8.43 (*SD*= 2.29), mean social support of 40.27 (*SD*= 8.28), mean general perceived quality of life of 3.44 (*SD*= 0.72) and mean general perceived health of 3.34 (*SD*= 0.83) (Table 1).

**Table 1.** Description of older adults’ functional dependence, family functioning, social support and quality of life – Heroica Matamoros, Tamaulipas, Mexico, 2019.

Variables	Mean	SD	CI 95%	
			Lower limit	Upper limit
Basic Activities of Daily Living	94.21	13.28	91.97	95.77
Instrumental Activities of Daily Living	6.39	2.29	6.06	6.67
Family functioning	8.43	2.24	8.09	8.75
Social support	40.27	8.28	39.26	41.40
Perceived quality of life	3.44	0.72	3.34	3.57
Perceived health	3.34	0.83	3.21	3.47
Physical quality of life	60.52	14.88	58.51	62.64
Phycological quality of life	67.43	12.44	65.87	69.24
Social quality of life	61.26	18.27	58.80	63.89
Environmental quality of life	55.12	12.44	53.25	57.17

SD = Standard Deviation; 95% CI = 95% Confidence Interval.

The correlation between variables showed a positive and statistically significant relationship between BADL, IADL, family functioning and social support with all dimensions of quality of life. The correlation between BADL with physical quality of life (*r*s = 0.467, *p*< 0.001) stood out. Likewise, the correlations between IADL with physical (*r*s = 0.426, *p*< 0.001) and psychological (*r*s = 0.418, *p*< 0.001) quality of life stood out. Finally, the correlation between social support and social quality of life (*r*s = 0.477, *p*< 0.001) stood out (Table 2).

**Table 2.** Relationship between functional dependence, family functioning and social support with older adults’ quality of life – Heroica Matamoros, Tamaulipas, Mexico, 2019.

	Perceived quality of life	Perceived health	Physical	Psychological	Social	Environmental
BADL	0.083	0.180**	0.467**	0.352**	0.221**	0.161*
IADL	0.204**	0.267**	0.426**	0.418**	0.226**	0.279**
Family functioning	0.264**	0.127	0.197**	0.255**	0.268**	0.349**
Social support	0.265**	0.225**	0.255**	0.332**	0.477**	0.351**

*p< 0.05 and **p< 0.01; BADL = Basic Activities of Daily Living; IADL = Instrumental Activities of Daily Living.

In the multiple linear regression analysis, a general multivariate model was used, where the covariates were age, sex, ABDL and IADL dependence, family function and social support. For the dependent variables, individuals’ general perceived quality of life and their health and quality of life dimensions were used. Once the model was made, a discriminant analysis was carried out with Wilks’ Lambda statistic, where it was observed that sex (Λ = 0.967, *p*= 0.370) and age (Λ = 0.941, *p*= 0.066) did not present statistical significance within the model, so it was decided to eliminate them (Table 3).

**Table 3.** Discriminant analysis with Wilks’ Lambda for general linear model – Heroica Matamoros, Tamaulipas, Mexico, 2019.

	Λ	F	DF of the hypothesis	DF of the error	p
Intersection	0.943	1.936	6.00	193.00	0.077
Sex	0.967	1.090	6.00	193.00	0.370
Age	0.941	2.010	6.00	193.00	0.066
BADL	0.906	3.347	6.00	193.00	0.004
IADL	0.883	4.273	6.00	193.00	0.000
Family function	0.935	2.248	6.00	193.00	0.040
Social support	0.736	11.526	6.00	193.00	0.000

Λ = Wilk’s Lambda; BADL = Basic Activities of Daily Living; IADL = Instrumental Activities of Daily Living; DF = Degrees of Freedom; F = F-test (explanatory of independent and dependent variables); *p* = statistical significance.

After the previous analysis, we proceeded to perform the inter-subject effects test, where it was observed that all corrected models are statistically significant. Individually, BADL were significant for some dimensions, among which physical quality of life stands out (*F* [1, 200] = 24.42, *p*< 0.001), indicating that it has an effect on it.

Regarding IADL, this variable had an effect on some dimensions, among which psychological quality of life (*F* [1, 200] = 20.64, *p*< 0.001) and health satisfaction (*F* [1, 200] = 10.48, *p*= 0.001) stand out. In the case of family function, this was significant only for perceived quality of life (*F* [1, 200] = 4.04, *p*= 0.046), contrary to social support, which was significant for all dependent variables except for perceived quality of life (Table 4).

**Table 4.** Test of effects of inter-subjects of covariates and dependent variables – Heroica Matamoros, Tamaulipas, Mexico, 2019.

Origin	Dependent variable	Type III sums of squares	DF	Mean square	F	*p*
Corrected model	Perceived quality of life	10.428	4	2.607	5.421	0.000
	Perceived health	18.648	4	4.662	7.553	0.000
	Physical	15418.344	4	3854.586	25.900	0.000
	Psychological	11491.521	4	2872.880	28.554	0.000
	Social	23662.199	4	5915.550	26.621	0.000
	Environmental	7608.518	4	1902.129	15.860	0.000
Intersection	Perceived quality of life	13.589	1	13.589	28.259	0.000
	Perceived health	8.761	1	8.761	14.193	0.000
	Physical	26.394	1	26.394	.177	0.674
	Psychological	1116.902	1	1116.902	11.101	0.001
	Social	229.981	1	229.981	1.035	0.310
	Environmental	758.420	1	758.420	6.324	0.013
BADL	Perceived quality of life	0.002	1	0.002	0.005	0.943
	Perceived health	0.137	1	0.137	0.223	0.637
	Physical	3634.128	1	3634.128	24.418	0.000
	Psychological	527.263	1	527.263	5.241	0.023
	Social	1167.665	1	1167.665	5.255	0.023
	Environmental	125.873	1	125.873	1.050	0.307
IADL	Perceived quality of life	1.508	1	1.508	3.136	0.078
	Perceived health	6.471	1	6.471	10.483	0.001
	Physical	997.290	1	997.290	6.701	0.010
	Psychological	2076.589	1	2076.589	20.640	0.000
	Social	224.547	1	224.547	1.010	0.316
	Environmental	567.256	1	567.256	4.730	0.031
Family function	Perceived quality of life	1.941	1	1.941	4.036	0.046
	Perceived health	0.208	1	0.208	0.336	0.563
	Physical	0.648	1	0.648	0.004	0.947
	Psychological	177.899	1	177.899	1.768	0.185
	Social	58.164	1	58.164	0.262	0.609
	Environmental	296.293	1	296.293	2.471	0.118
Social support	Perceived quality of life	0.780	1	0.780	1.623	0.204
	Perceived health	3.075	1	3.075	4.982	0.027
	Physical	1881.059	1	1881.059	12.639	0.000
	Psychological	1502.159	1	1502.159	14.930	0.000
	Social	14089.274	1	14089.274	63.404	0.000
	Environmental	2166.388	1	2166.388	18.064	0.000
Error	Perceived quality of life	96.177	200	0.481		
	Perceived health	123.450	200	0.617		
	Physical	29765.454	200	148.827		
	Psychological	20122.300	200	100.612		
	Social	44442.815	200	222.214		
	Environmental	23986.090	200	119.930		
Total	Perceived quality of life	2538.000	205			
	Perceived health	2431.000	205			
	Physical	796096.939	205			
	Psychological	963958.333	205			
	Social	837430.556	205			
	Environmental	654472.656	205			
Total corrected	Perceived quality of life	106.605	204			
	Perceived health	142.098	204			
	Physical	45183.798	204			
	Psychological	31613.821	204			
	Social	68105.014	204			
	Environmental	31594.607	204			

BADL = Basic Activities of Daily Living; IADL = Instrumental Activities of Daily Living; DF = Degrees of Freedom; F = F-test (explanatory of independent and dependent variables); *p* = statistical significance.

According to the generalized linear model, for the quality of life’s response vector, only the family function covariate (Λ = 0.05, *F* [1, 200] = 4.04, *p*= 0.046) presented a predictive effect, which indicates that the ability to cope with situations of family stress, mutual support and affective relationships act as a protective factor on older adults’ quality of life. For the health satisfaction’s response vector, the IADL covariate stands out for its predictive effect (Λ = 0.10, *F* [1, 200] = 10.48, *p*= 0.001), which indicates that while older adults are able to perform activities such as preparing food, home maintenance and care and communicate by various means, this acts as a protective effect to feel satisfied about their health.

According to the physical health dimension’s response vector, BADL (Λ = 0.40, *F* [1, 200] = 24.41, *p*< 0.001) and social support (Λ = 0.44, *F* [1, 200] = 12.64, *p*< 0.001) stand out for their predictive effect. This indicates that, if older adults are able to perform hygiene, clothing and feeding activities, and also perceive external support, this represents a protective factor that influences the perception of a good physical quality of life.

Regarding the psychological health dimension’s response vector, it could be observed that the predictive effect of IADL (Λ = 1.82, *F* [1, 200] = 20.64, *p*< 0.001) and social support (Λ = 0.39, *F* [1, 200] = 14.93, *p*< 0.001) stands out. This indicates that, if older adults are able to perform IADL as mentioned above, and have support from the family and society, they become a protective factor for a quality of life in the psychological field.

Similarly, for social and environmental health dimensions’ response vectors, the predictive effect of social support (Λ = 1.19, *F* [1, 200] = 63.40, *p*< 0.001 and Λ = 0.47, *F* [1, 200] = 18.06, *p*< 0.001) stands out, which indicates that family and social support in older adults is a protective factor that influences the perception of their quality of social and environmental life (Table 5).

**Table 5.** Generalized linear model of perceived quality of life, health satisfaction and quality of life dimensions with Basic and Instrumental Activities of Daily Living, family function and social support of older adults – Heroica Matamoros, Tamaulipas, Mexico, 2019.

Dependent variable	Parameter	B	p	95% Confidence Interval	
				L_l_	U_l_
Quality of life	Intersection	2.357	0.000	1.483	3.231
	BADL	0.000	0.943	–0.009	0.009
	IADL	0.049	0.078	–0.006	0.104
	Family function	0.053	0.046	0.001	0.105
	Social support	0.009	0.204	–0.005	0.023
Health satisfaction	Intersection	1.893	0.000	0.902	2.883
	BADL	0.002	0.637	–0.008	0.013
	IADL	0.102	0.001	0.040	0.164
	Family function	–0.017	0.563	–0.076	0.042
	Social support	0.018	0.027	0.002	0.033
Physical	Intersection	–3.285	0.674	–18.667	12.097
	BADL	0.402	0.000	0.242	0.563
	IADL	1.265	0.010	0.301	2.229
	Family function	0.031	0.947	–0.882	0.943
	Social support	0.436	0.000	0.194	0.678
Psychological	Intersection	21.369	0.001	8.722	34.016
	BADL	0.153	0.023	0.021	0.285
	IADL	1.826	0.000	1.033	2.618
	Family function	0.506	0.185	–0.244	1.257
	Social support	0.390	0.000	0.191	0.589
Social	Intersection	–9.697	0.310	–28.492	9.099
	BADL	0.228	0.023	0.032	0.424
	IADL	0.600	0.316	–0.577	1.778
	Family function	–0.289	0.609	–1.405	0.826
	Social support	1.194	0.000	0.898	1.489
Environmental	Intersection	17.609	0.013	3.801	31.417
	BADL	0.075	0.307	–0.069	0.219
	IADL	0.954	0.031	0.089	1.819
	Family function	0.653	0.118	–0.166	1.473
	Social support	0.468	0.000	0.251	0.685

CI = Confidence Interval; Ll= lower limit; Ul= Upper limit; BADL = Basic Activities of Daily Living; IADL = Instrumental Activities of Daily Living.

## DISCUSSION

The data analyzed in the study show that perceived quality of life in older adults depends on factors such as having functional independence, an adequate family function and a positive perceived health. It was observed that the lower the dependence to perform BADL and IADL, the higher the quality of life in all dimensions. This result agrees with that reported by other authors who refer that dependence is associated with alteration in quality of life^(6,18)^. This relationship can be explained by other authors who indicate that functional capacity, being one of the main determinants of health, affecting the development of activities of daily living with an impact on older adults’ quality of life^(19)^.

Therefore, it is understood that to be independent requires physical skills. Although these can decrease with age, they are not part of aging, so it is important to promote healthy aging and that nursing professionals continuously assess the levels of functional dependence, in order to identify this problem in a timely manner and apply nursing interventions that allow functional dependence to be delayed as much as possible in the search for aging with quality of life.

In the study of quality of life, it is important to consider older adults’ support networks, such as their family and social context. Older adults as social beings need family and social relationships that allow them to feel protected and accepted. Scientific literature indicates that being and feeling part of a social group impacts older adults’ general well-being^(20)^, so it is considered that, if older adults perceive social support, their quality of life will be favored. Research results have shown empirically that perceived social support is associated with higher quality of life in the old population^(12–21)^.

In this study, it was found that family functioning is related to quality of life, similar to that reported by other authors^(10)^, which suggests that when older adults feel loved and supported by their family, they perceive the support and protection necessary to meet their needs. On the contrary, when older adults live in a dysfunctional family, their mood can be altered, which leads to social and physical problems that culminate in alteration in quality of life^(2)^.

Regarding the social context, it was observed that the greater social support, the higher the quality of life. These results resemble those obtained in other research^(18,22)^, where results show that there is a positive and significant correlation between social support and quality of life, which means that the better the context in which older adults develop and the support they receive, the greater their perceived quality of life.

In the linear regression analysis, it was observed that BADL had a significant effect on the physical, psychological and social quality of life dimensions. Meanwhile, IADL resulted with significant effect on the physical, psychological and environmental quality of life dimensions. The effect found between BADL and IADL on quality of life dimensions coincides with that reported by other authors who have reported statistical significance between independence and quality of life^(23,24)^.

Finally, it was identified that family functioning has a significant effect on older adults’ quality of life; for this reason and according to what has been reported by other authors, the family environment as the first support network of older adults is important for quality of life^(25)^. In this regard, the important work of nursing professionals from the first contact with older adults is highlighted, where it is recommended to include BADL, IADL and family functionality, aiming at applying nursing interventions on the variables that have been proven to impact quality of life.

## CONCLUSIONS

Older adults’ quality of life depends on factors such as having functional independence, adequate family function and a positive perceived health. In the physical domain, having independence for BADL and having adequate social support improve quality of life. In the psychological domain, it was found that being independent for BADL and social support improve quality of life.

By presenting a decrease in independence in for IADL, perceived quality of life also decreases. Finally, it was verified that having a good social support is a protective factor that influences the perception of their quality of life in the social and environmental domains.

## ASSOCIATE EDITOR

Marcia Regina Martins Alvarenga
